# Metal–Organic Gel Leading to Customized Magnetic-Coupling Engineering in Carbon Aerogels for Excellent Radar Stealth and Thermal Insulation Performances

**DOI:** 10.1007/s40820-023-01255-7

**Published:** 2023-12-04

**Authors:** Xin Li, Ruizhe Hu, Zhiqiang Xiong, Dan Wang, Zhixia Zhang, Chongbo Liu, Xiaojun Zeng, Dezhi Chen, Renchao Che, Xuliang Nie

**Affiliations:** 1 Key Laboratory of Jiangxi Province for Persistent Pollutants Control and Resources Recycle, Nanchang Hangkong University, Nanchang, 330063 People’s Republic of China; 2 School of Environmental and Chemical Engineering, Nanchang Hangkong University, Nanchang, 330063 People’s Republic of China; 3 Laboratory of Advanced Materials, Department of Materials Science and Collaborative Innovation Center of Chemistry for Energy Materials and Collaborative Innovation Center of Chemistry for Energy Materials, Fudan University, Shanghai, 200438 People’s Republic of China; 4 School of Materials Science and Engineering, Jingdezhen Ceramic University, Jingdezhen, 333403 People’s Republic of China; 5 College of Chemistry and Materials, Jiangxi Agricultural University, Nanchang, 330045 People’s Republic of China

**Keywords:** Metal–organic gels, Heterometallic magnetic coupling, Radar stealth, Thermal insulation, Computer simulation technology

## Abstract

Metal–organic gel (MOG) derived composites are promising multi-functional materials due to their alterable composition, identifiable chemical homogeneity, tunable shape, and porous structure. Herein, stable metal–organic hydrogels are prepared by regulating the complexation effect, solution polarity and curing speed. Meanwhile, collagen peptide is used to facilitate the fabrication of a porous aerogel with excellent physical properties as well as the homogeneous dispersion of magnetic particles during calcination. Subsequently, two kinds of heterometallic magnetic coupling systems are obtained through the application of Kirkendall effect. FeCo/nitrogen-doped carbon (NC) aerogel demonstrates an ultra-strong microwave absorption of − 85 dB at an ultra-low loading of 5%. After reducing the time taken by atom shifting, a FeCo/Fe_3_O_4_/NC aerogel containing virus-shaped particles is obtained, which achieves an ultra-broad absorption of 7.44 GHz at an ultra-thin thickness of 1.59 mm due to the coupling effect offered by dual-soft-magnetic particles. Furthermore, both aerogels show excellent thermal insulation property, and their outstanding radar stealth performances in J-20 aircraft are confirmed by computer simulation technology. The formation mechanism of MOG is also discussed along with the thermal insulation and electromagnetic wave absorption mechanism of the aerogels, which will enable the development and application of novel and lightweight stealth coatings.

**Supplementary Information:**

The online version contains supplementary material available at 10.1007/s40820-023-01255-7.

## Highlights

- Fe^3+^, Co^2+^, H_3_BTC, and collagen peptide are used to achieve a one-step assembly of stable FeCo-MOG/CP by manipulating the complexation effect and solution polarity.
- By optimizing pyrolysis, two kinds of nitrogen-doped carbon aerogels loaded with virus-shaped and nanospherical magnetic particles are obtained.
- FeCo/Fe_3_O_4_/NC and FeCo/NC aerogels exhibit excellent electromagnetic wave absorbing and radar stealth performances.

**Supplementary Information:**

The online version contains supplementary material available at 10.1007/s40820-023-01255-7.

## Graphical abstract

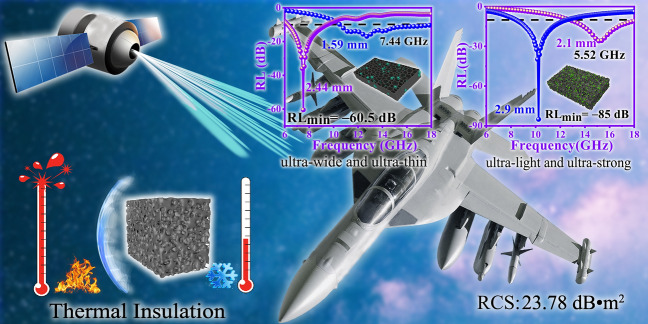

## Introduction

Electromagnetic waves (EMWs) are widely used in the fields of wireless communication and radar detection [1, 2]. 5G and 6G communication technologies cause radiation pollution problems in civilian fields, and the rapid development of detection technologies has led to the decline of weapon capabilities in the military, thus rendering electromagnetic wave absorbing (EMWA) materials a research hotspot [2–5].

Structure and composition are two crucial factors for fabricating excellent EMWA materials [6–8]. Owing to their lightweight characteristics, broad EMWA bands, and multi-functionality, carbon-based aerogels are considered one of the most promising EMWA materials [9–11]. The addition of magnetic particles into carbon aerogels leads to a better EMWA performance due to improved impedance matching [12, 13]. In general, conventional magnetic carbon-based aerogels are prepared either via mechanical mixing or utilizing the electrostatic effect of magnetic particles and gel precursor; however, the magnetic particles in these aerogels are prone to agglomeration, which limits their performance [14, 15].

Metal organic frameworks (MOFs) with periodic structures are considered promising EMWA precursors because their derivatives possess alterable dielectric/magnetic components as well as uniform structures wherein magnetic particles do not accumulate [16]. Hence, metal–organic gel (MOG) can be used as a precursor to obtain lightweight and porous aerogels that allow a uniform distribution of magnetic particles in carbon matrix; however, the high capillary force of MOG makes it difficult to form stable aerogels during the drying process [17]. Collagen peptide (CP) with its abundant –NH_2_ and –OH groups can be closely interweaved with the fundamental skeleton of MOG through weak molecular interaction forces, which consolidates the gel structure of MOG [18].

Here, we fabricate a stable FeCo-MOG by adding CP and manipulating the solution polarity and complexation force of metal ions with 1,3,5-benzenetricarboxylic acid (H_3_BTC) [19]. The cross-polarization and electromagnetic coupling of CP with a micro-helical structure make the material less sensitive to the frequency of EMWs, which leads to wide EMWA bands [20]. After carbonization of FeCo-MOG/CP, an aerogel is obtained that not only possesses the advantages of MOF derivatives, but also the physical properties of carbon aerogels such as thermal insulation. More interestingly, two kinds of magnetic carbon aerogels have been obtained by simply adjusting the pyrolysis process, which exhibit excellent EMWA performance. The nano-sized FeCo at a calcination rate of 2 °C min^−1^ shows small size effects. In particular, at a rate of 5 °C min^−1^, two types of soft magnetic particles (FeCo/Fe_3_O_4_) are simultaneously and evenly imbedded in carbon aerogel, which forms a virus-shaped structure [21].

Magnetic metals, metal oxides, and alloys are the most widely known magnetic materials. FeCo alloy with its high saturation magnetization has been widely used in the design of EMWA materials. Yang et al. successfully prepared a porous succulent-like FeCo alloy via a facile hydrothermal method [22]. Owing to its high permeability, the FeCo alloy achieved a minimum reflection loss (*RL*_*min*_) of − 53.81 dB and an effective absorption bandwidth (*f*_*e*_, *RL* ≤  − 10 dB) of 5.68 GHz. However, the conductivity of FeCo is high, and EMWs tend to easily reflect on the FeCo surface. Fe_3_O_4_, a magnetic semiconductor material with a narrow bandgap, is considered a good raw material for constructing broadband EMWA materials. Zhi et al. prepared a core–shell bilayer coupling material loaded with Fe_3_O_4_, which exhibited an *f*_*e*_ of 6.88 GHz at 2.5 mm-thickness [23]. Therefore, it is sensible to utilize the high permeability of FeCo and the low dielectric constant of Fe_3_O_4_ to balance their dielectric electromagnetic characteristics and construct a magnetic coupling network with enhanced EMWA performance.

In addition, the similar atomic radii of N and C allow N to be easily doped into C. Moreover, the C atoms near N have a higher charge density, resulting in better electrical conductivity. Therefore, N-doping greatly enhances the conduction and polarization loss of EMWA materials, which improves their EMWA capability [24–26]. Sun et al. doped N into reduced graphene oxide, which effectively improved the electronic transmission and polarization and increased *RL*_*min*_ to − 52 dB under an extremely low loading of 2 wt%. However, *f*_*e*_ was only 3.9 GHz at a thickness of 3.8 mm due to a lack of magnetic loss. Guo et al. in situ grew magnetic materials on honeycomb-like nitrogen-doped carbon (NC), wherein *f*_*e*_ achieved 5.36 GHz owing to an electromagnetic synergistic effect [27].

Accordingly, by utilizing MOG prepared via a one-step assembly, we ensure that soft magnetic particles are evenly distributed in the NC aerogel, which reduces the density of the material, enhances thermal insulation performance, and significantly improves impedance matching and EMWA performance. This work provides insights into lightweight broadband EMWA materials and offers a novel method for the preparation of magnetic carbon aerogels.

## Experimental Section

Fe(NO_3_)_3_·9H_2_O, Co(NO_3_)_2_·6H_2_O, and 1,3,5-benzenetricarboxylic acid (H_3_BTC) were purchased from Aladdin Biochemical Technology Co., Ltd. (Shanghai, China). CP was purchased from Shanghai Dingfen Chemical Technology Co., Ltd. (Shanghai, China). All chemicals were used without further purification.

### Syntheses of FeCo-MOG/CP

First, 1 g H_3_BTC was dissolved in a solution comprising 10 mL ethanol and 3 mL deionized water via vigorous ultrasonic stirring; the resulting solution was named A. Second, 0.75 g Fe(NO_3_)_3_·9H_2_O, 0.55 g Co(NO_3_)_2_·6H_2_O, and 1 g CP were dissolved in 5 mL deionized water; this solution was named B. Finally, solution B was poured into solution A and stirred. After a few minutes, the FeCo-MOG/CP was formed. To obtain dry FeCo-MOG/CP, the hydrogel is demolded, placed on an evaporation dish, and then freeze-dried for 48 h.

### Syntheses of FeCo/NC and FeCo/Fe_3_O_4_/NC

Dry FeCo-MOG/CP was annealed for 2 h in a tube furnace under the protection of N_2_ at 600 and 700 °C, and the FeCo/NC and FeCo/Fe_3_O_4_/NC aerogels were obtained at a heating rate of 2 and 5 °C min^−1^, respectively.

### Characterization

The sample morphologies were surveyed using field emission scanning electron microscopy (FE-SEM; FEI Nova Nano SEM450) and TEM (JEOL, JEM-2100F and Talos F2000X). The phase structures of the samples were characterized using powder X-ray diffraction (PXRD; Bruker D8 Advance A25) under CuKα radiation. The surface compositions were analyzed using X-ray photoelectron spectroscopy (XPS; Thermo Fischer ESCALAB Xi+). The pictures depicting the thermal insulation performance of aerogels were captured using a thermal imaging camera (TVS-2000MK). The saturation magnetization of the samples was inspected using a physical property measurement system (PPMS; Quantum Design Dyna COOL). Meanwhile, graphitization degree was investigated using Raman spectroscopy (Lab RAM HR800) from 800 to 2000 cm^−1^ under 532 nm laser excitation. The electromagnetic characteristics of specimens in the range of 2–18 GHz were analyzed using a vector network analyzer (Agilent PNA N5224A, coaxial line method). The thermal diffusivity values were obtained by a thermal constant analyzer (Hot Disk TPS2500S) at room temperature. The radar cross sections (RCS) are simulated using computer simulation technology (CST; Studio Suite 2019). All the relevant details can be found in the supporting information (SI).

## Results and Discussion

### Preparation and Forming Mechanism of Gels

**Fig. 1:**
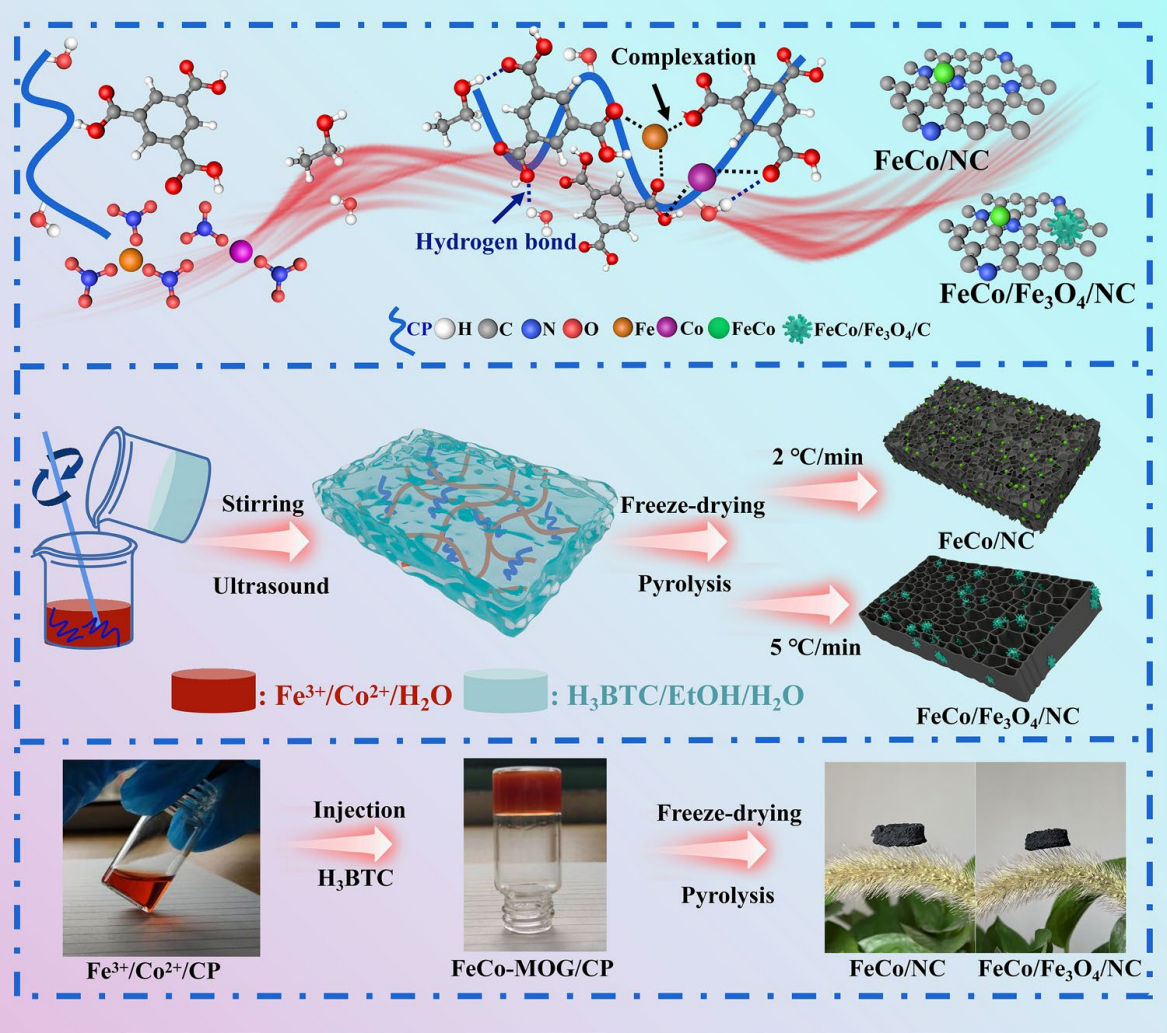
Schematic for the synthesis of FeCo/NC and FeCo/Fe_3_O_4_/NC

To ensure that magnetic particles are not easily agglomerated, novel magnetic carbon aerogels have been prepared for the first time using MOG through the sol–gel and high-temperature pyrolysis methods. The synthetic route of this work is illustrated in Fig. 1. Fe^3+^ and Co^2+^ are mixed with H_3_BTC and CP adequately under ultrasonic stirring, and the resulting solution is made to stand for a few minutes to form the FeCo-MOG/CP via supramolecular self-assembly. Subsequently, the hydrogel is subjected to freeze-drying and the magnetic NC aerogels are obtained by carbonizing dry gel in a N_2_ atmosphere. The specific preparation methods have been described in the experimental section, and the formation mechanism of FeCo-MOG is as follows. The carboxyl groups of H_3_BTC can coordinate with metal ions in multiple directions. Further, under the polar interaction of solution, numerous metal-carboxylate coordination bonds are stretched, which generates micelles and traps the solvent molecules to form a metal-carboxylate gel network. It is worth noting that the self-assembly of hydrogels is influenced by many factors. For instance, the greater the metal ion and ligand concentrations, the faster the gel forms; meanwhile, pure alcohol results in rapid gel formation, and the alcohol-water solvent mixture is beneficial for mild gel formation and helps CP molecules to cooperate effectively. CP exhibits negative electrical charge and hydrophilic properties due to its rich functional groups such as carboxyl, amino, and hydroxyl groups. In summary, the hydrophobic interaction makes the hydrophobic part aggregate inward and exposes the hydrophilic groups, which in turn increases electrostatic exclusion, facilitates hydration shell due to the solvent effect, and enhances the steric hindrance and mechanical resistance among the particles [28]. Consequently, this leads to either a reduction or complete elimination of the aggregation and precipitation of the particles. Thus, a balance between hydrophilicity and hydrophobicity is achieved and a stable MOG system is formed.

By employing different pyrolysis processes, two kinds of MOG derivatives are  obtained, which exhibit significant differences with regard to their composition and morphology. Cobalt ions are more easily reduced to metal than iron ions. Under high heating rate of 5 °C min^−1^ and a short heating period, both H_3_BTC and CP comprise plenty of oxygen. Thus, iron ions cannot be completely reduced and Fe_3_O_4_ is obtained. When the heating rate is lowered to 2 °C min^−1^ and calcination time is improved considerably, Co can promote the reduction of Fe_3_O_4_ to iron and reduced Fe combines with Co to form an FeCo alloy.

### Composition and Structure

**Fig. 2:**
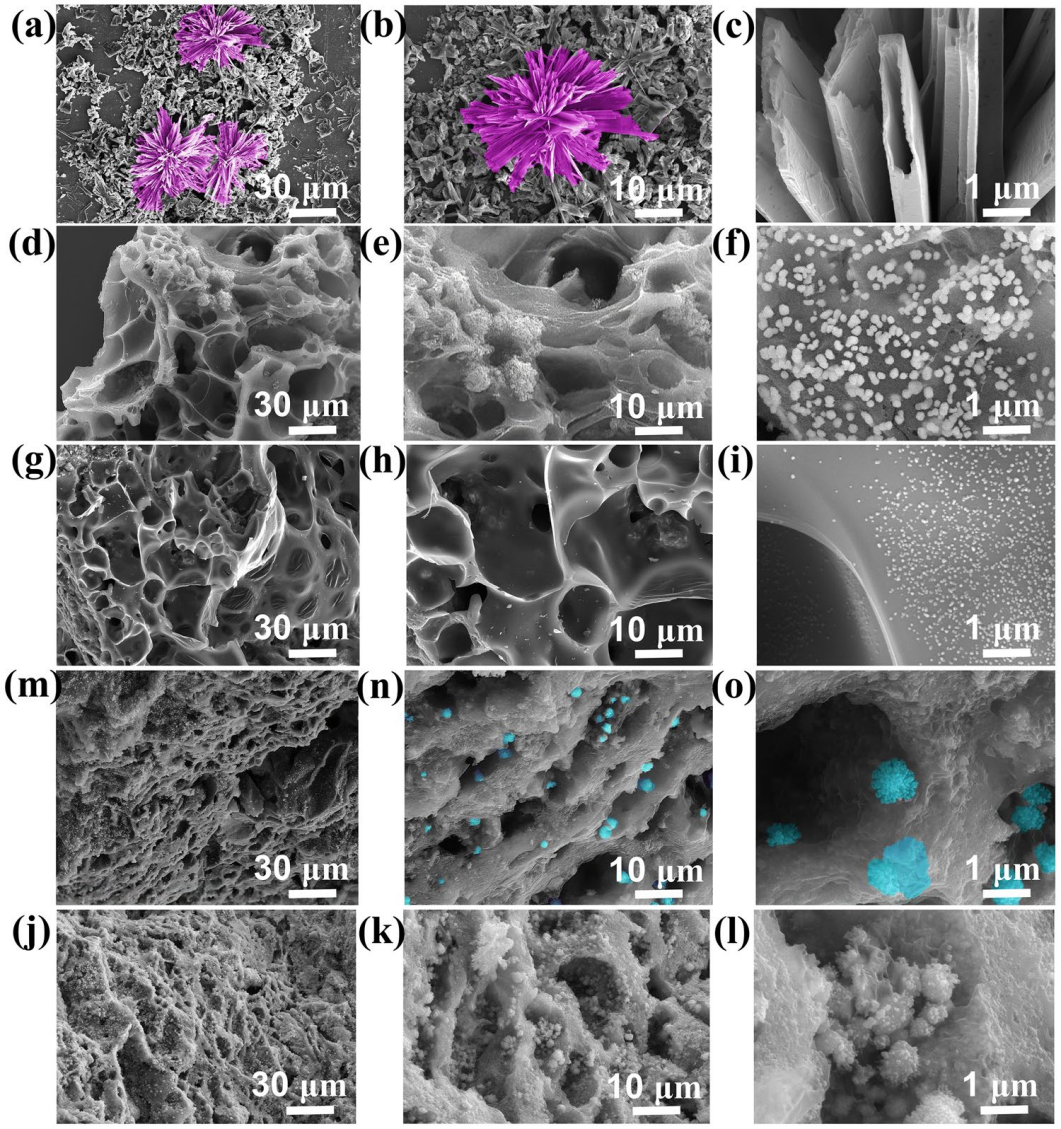
SEM images of **a**–**c** dry FeCo-MOG/CP, **d**–**f** FeCo/NC-600, **g**–**i** FeCo/NC-700, **j–l** FeCo/Fe_3_O_4_/NC-600, and **m**–**o** FeCo/Fe_3_O_4_/NC-700

The SEM images of FeCo-MOG/CP dry gel (Fig. 2a–c) exhibit the morphology of flowers; under a magnified view, it is observed that the petals comprise hollow fibers, which facilitate an increase in the porosity of FeCo/NC and FeCo/Fe_3_O_4_/NC aerogels. Figure 2d–i show that after carbonization, these aerogels maintain the porosity of their dry gel precursors. FeCo/NC-700 and FeCo/Fe_3_O_4_/NC-600 aerogels show a regular porous structure in which magnetic particles are evenly distributed. The FeCo/NC-700 aerogel obtained using a heat rate of 2 °C min^−1^ has a silky structure. Meanwhile, the FeCo/Fe_3_O_4_/NC-600 aerogel prepared at a heat rate of 5 °C min^−1^ shows a wafer-like structure, owing to the shorter calcination time and lower carbon loss. As shown in Fig. 2, FeCo/Fe_3_O_4_/NC-600 and FeCo/Fe_3_O_4_/NC-700 exhibit similar structures and so do FeCo/NC-600 and FeCo/NC-700. Meanwhile, pores become larger and nano-magnetic particles become smaller as the calcination temperature increases. At a higher magnification, virus-shaped magnetic nanoparticles of approximately 500 nm are firmly attached on the carbon matrix of FeCo/Fe_3_O_4_/NC-600. Owing to the Kirkendall effect, the diffusion rate of different atoms is not the same in the solid state [29]. Furthermore, the magnetic particles and carbon atoms nonuniformly shift outward due to different thermal vibrations at high temperatures, which results in the formation of virus-like particles. Moreover, FeCo with its smaller size is uniformly dispersed in the FeCo/NC-700 aerogel due to the dissociation of the micrometer-scale virus-shaped particles at a lower heating rate.

**Fig. 3:**
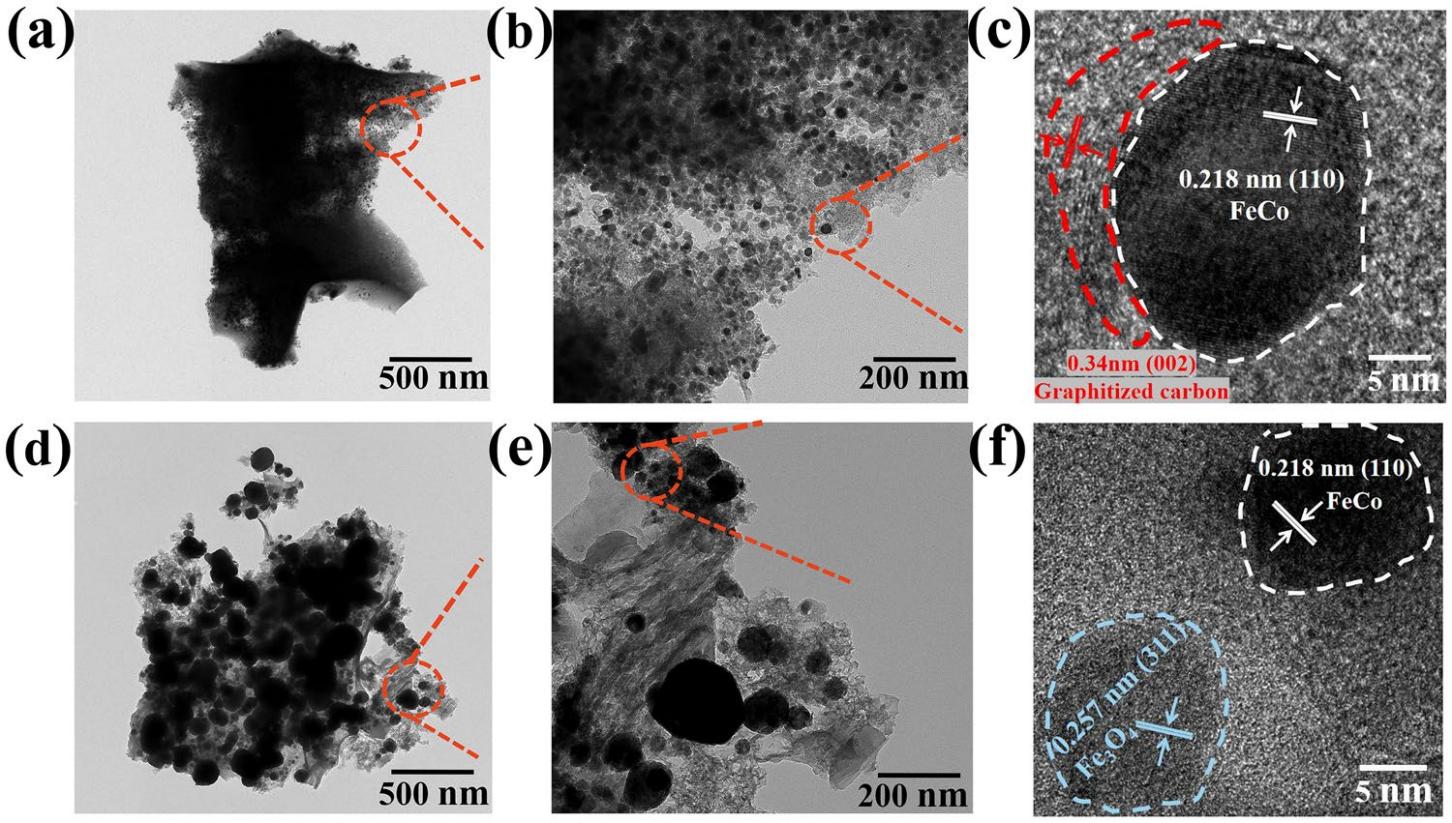
TEM images of **a**–**c** FeCo/NC-700, and** d**–**f** FeCo/Fe_3_O_4_/NC-600

Transmission electron microscopy (TEM) images of the aerogels are illustrated in Fig. 3. The FeCo particles of FeCo/NC-700 are dispersedly distributed in the NC matrix with an average size of approximately 10 nm, while the sizes of FeCo and Fe_3_O_4_ particles in the FeCo/Fe_3_O_4_/NC-600 aerogel range from 10 to 200 nm. The high-resolution TEM (HRTEM) image of FeCo/NC-700 shows that the lattice spacing of 0.218 nm corresponds to the (110) lattice plane of FeCo particles, while that of 0.34 nm corresponds to the (002) lattice plane of graphitized carbon. The (110) lattice plane of FeCo can also be observed for FeCo/Fe_3_O_4_/NC-600, which also exhibits a lattice spacing of 0.257 nm corresponding to the (311) crystal plane of Fe_3_O_4_. The selected area electron diffraction image indicates that the carbon lattice fringe of FeCo/NC-700 exhibits a higher graphitization degree than that of FeCo/Fe_3_O_4_/NC-600, and the magnetic particles are separated by carbon. The high-angle annular dark-field scanning TEM and element distribution maps (Fig. S1) show the uniform distribution of nitrogen in the carbon matrix, which proves the interweaving of CP molecules with metal–organic micelles. Meanwhile, similar Fe and Co distributions prove that FeCo alloy has been successfully prepared. As shown in Fig. S1, the elemental mapping images of FeCo/NC-700 and FeCo/Fe_3_O_4_/NC-600 both imply that C, N, O, Fe, and Co coexist. All elements are uniformly distributed in FeCo/NC-700, indicating that small FeCo particles are evenly embedded in the NC matrix. For FeCo/Fe_3_O_4_/NC-600, the signal intensity of O is significantly enhanced after the introduction of Fe_3_O_4_. The large spherical particles shown in yellow and red correspond to O and Fe, respectively; these particles are more pronounced because Fe_3_O_4_ is larger than FeCo. This result can prove that Fe_3_O_4_ is produced by increasing the heating rate.

**Fig. 4:**
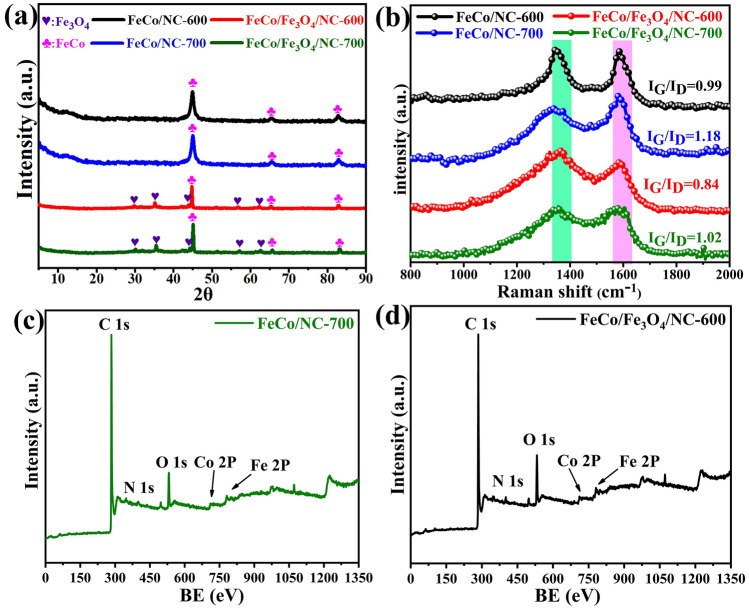
**a** XRD patterns; **b** Raman spectra; XPS spectra of **c** FeCo/Fe_3_O_4_/NC-600, and **d** FeCo/NC-700

The phase structures of the samples are investigated using X-ray diffraction (XRD). As shown in Fig. S2, the diffraction peaks of FeCo-MOG/CP are sharp and intense and no impurity peaks are observed, which confirms the high purity of the precursors. Figure 4a shows that all samples illustrate distinct diffraction peaks associated with the (110), (200), and (211) planes of FeCo (JCPDS#49-1568). The XRD patterns of the FeCo/Fe_3_O_4_/NC aerogel demonstrate five additional peaks at 30.1°, 35.4°, 37.1°, 56.9°, and 62.6°, which are attributed to the (220), (311), (222), (511), and (440) crystalline planes of Fe_3_O_4_ according to the JCPDS card number 85-1436, respectively. More specifically, the peaks of Fe_3_O_4_ are substantially weaker than those of FeCo, thus demonstrating that only a small amount of Fe_3_O_4_ has been generated. Figure 4b shows the Raman spectra of samples, which indicates that the graphitization degree of samples increases with a rise in carbonization temperature and time.

Figure 4c–d demonstrates the X-ray photoelectron spectroscopy (XPS) images, which are used to examine the chemical valence states of the samples. The survey scans demonstrate that FeCo/NC-700 and FeCo/Fe_3_O_4_/NC-600 both comprise C, N, O, Fe, and Co elements. Figure S3 shows that the high-resolution spectra of FeCo/NC-700 and FeCo/Fe_3_O_4_/NC-600 are similar. For both samples, the C 1*s* spectrum shows four peaks corresponding to C–C/C=C, C–N, C–O, and C=O, which reveal that some functional groups in the precursor are retained [13]. High-resolution spectrum of N 1*s* reveals the formation of pyridinic, pyrrolic, and graphitic nitrogen, thus confirming the successful doping of N [30]. The Co 2*p* spectrum shows two peaks of 2*p*_1/2_ and 2*p*_3/2_, thereby indicating the presence of Co^0^. As shown in Fig. S3e, the high-resolution O 1*s* spectrum of FeCo/NC aerogel exhibits four peaks at 530.1, 531.6, 531.8 and 533.6 eV, corresponding to O=C, O–C, O–Co and O–Fe bands, respectively; here, the oxygen content is sourced from the NC matrix in the form of O–C and O=C, while oxygen also exists as O–Fe and O–Co after the oxidation of surface FeCo alloy. Besides, Fig. S3f exhibits a similar high-resolution O 1*s* spectrum of FeCo/Fe_3_O_4_/NC aerogel. The Fe 2*p* spectra of both samples can be deconvoluted into multiple peaks corresponding to Fe^3+^, Fe^2+^, and Fe^0^ [31]. Owing to the existence of Fe_3_O_4_, the areas of peaks corresponding to Fe^2+^ and Fe^3+^ in FeCo/Fe_3_O_4_/NC-600 are greater than those in FeCo/NC-700. However, Fe^3+^ and Fe^2+^ are present in FeCo/NC-700 because external Fe is oxidized in air. Overall, XRD, XPS, and HRTEM demonstrate the successful preparation of FeCo/NC and FeCo/Fe_3_O_4_/NC aerogels.

### EMWA Performance

According to electromagnetic field theory, the EWMA capability of materials relies on their microstructures, relative permittivity (*ε*_r_ = *ε'-jε''*), and permeability (*μ*_r_ = *μ'-jμ''*), where *ε* and *μ* represent the dielectric and magnetic losses of EMWA materials, respectively [32, 33]. The ability to store and dissipate the electrical energy of EMWs is represented by *ε'* and *ε''* respectively, while the ability to store and dissipate EMW magnetic energy is represented by *μ'* and *μ''*, respectively [34, 35]. As shown in Fig. S4, the *ε'* values of all the samples decrease gradually with increasing frequency owing to a frequency dispersion behavior. Meanwhile, the *μ'* and *μ''* values of all the samples fluctuate smoothly within a small range. The dielectric loss tangent (*tanδ*_*ε*_) and magnetic loss tangent (*tanδ*_*μ*_) show the dielectric and magnetic loss capabilities of the material, respectively. As shown in Fig. S5, the *tanδ*_*μ*_ values of all aerogels are lower than their *tanδ*_*ε*_ values in the 2–18 GHz range. Therefore, dielectric loss plays a prominent role in EMW attenuation.

**Fig. 5:**
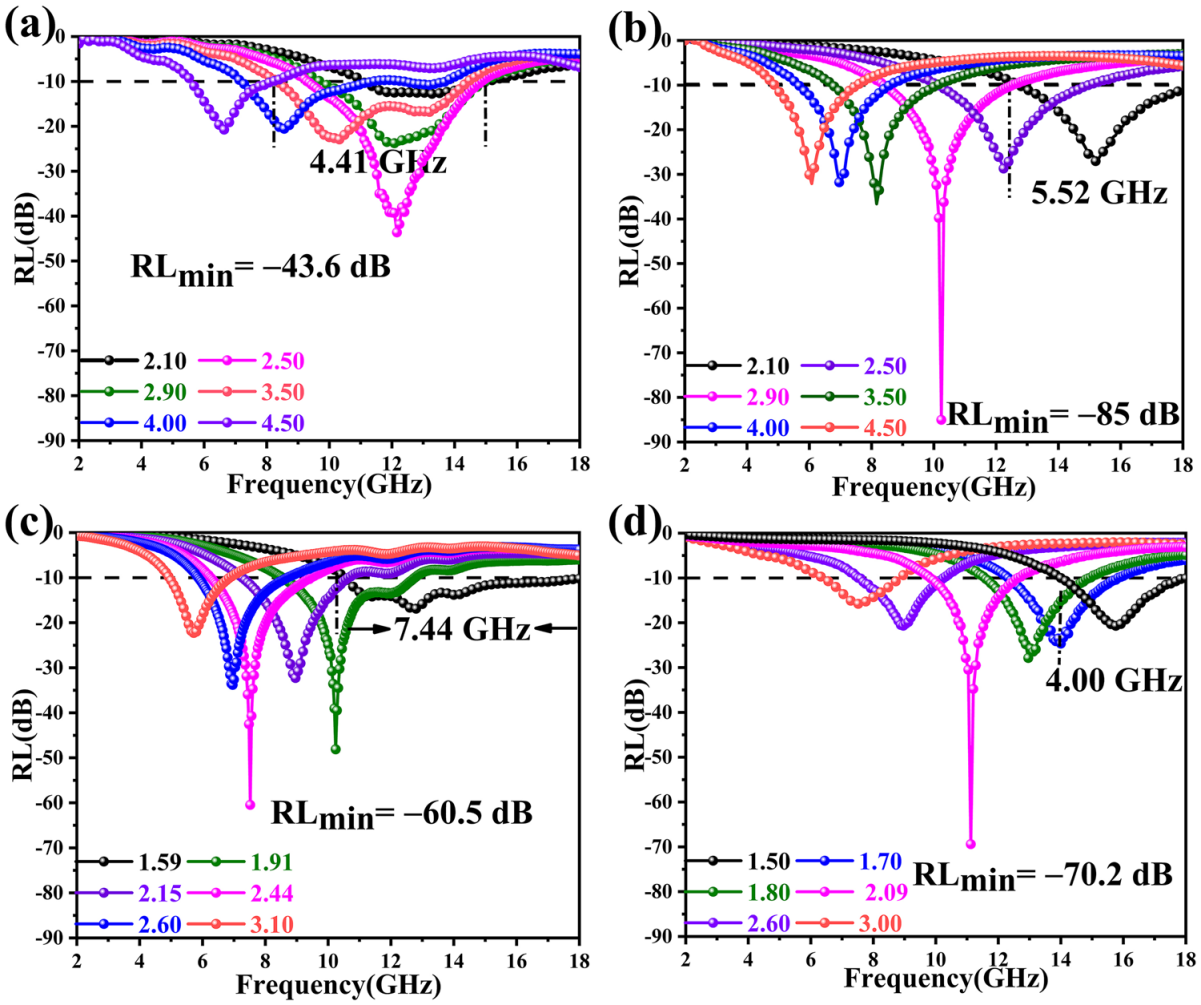
RL values of **a** FeCo/NC-600, **b** FeCo/NC-700, **c** FeCo/Fe_3_O_4_/NC-600, and **d** FeCo/Fe_3_O_4_/NC-700

Figure 5 shows the EMWA performances of all samples based on the transmission line theory (details regarding the calculation methods have been provided in the SI). When an undamaged 5 wt% aerogel sample is soaked in a 95 wt% paraffin solution, *RL*_*min*_ of FeCo/NC-700 is observed to be − 85 dB at 10.24 GHz and a thickness of 2.9 mm, while the broadest *f*_*e*_ equals 5.52 GHz at a thickness of 2.1 mm. However, to the best of our knowledge, the effect of heating rate on the components and EMWA performance has not been studied adequately. In the present study, dual-soft-magnetic nanoparticles of FeCo and Fe_3_O_4_ are obtained in the NC matrix by altering the heating rate, which leads to an ultra-wide absorption band. By adjusting the heating rate from 2 to 5 °C min^−1^, some of the Fe^3+^ ions are not fully reduced and FeCo/Fe_3_O_4_/NC is obtained. Owing to the rapid transfer of electrons between Fe^2+^ and Fe^3+^, Fe_3_O_4_ exhibits semiconducting characteristics and low coercivity, which helps improve impedance matching and further reduces the matching thickness of the material. For FeCo/Fe_3_O_4_/NC-600, the widest *f*_*e*_ of 7.44 GHz is observed at an ultra-thin thickness of 1.59 mm (10.56–18.00 GHz), thus exhibiting optimal usability. Meanwhile, the *RL*_*min*_ of FeCo/Fe_3_O_4_/NC-600 is -60.5 dB at 7.52 GHz and a thickness of 2.44 mm. As shown in Fig. S6, the *RL* peak frequency (*f*_*m*_) shifts to lower frequencies with increasing absorbed layer thicknesses (*t*_*m*_) and can be interpreted by the *λ/4* cancelation theory. The *f*_*m*_ and *t*_*m*_ values are calculated according to the following equation: ${\text{t}}_{{\text{m}}} = {\text{nc}}/\left( {4{\text{f}}_{{\text{m}}} \sqrt {\left| {\mu_{{\text{r}}} } \right|\left| {\varepsilon_{{\text{r}}} } \right|} } \right)$.

**Fig. 6:**
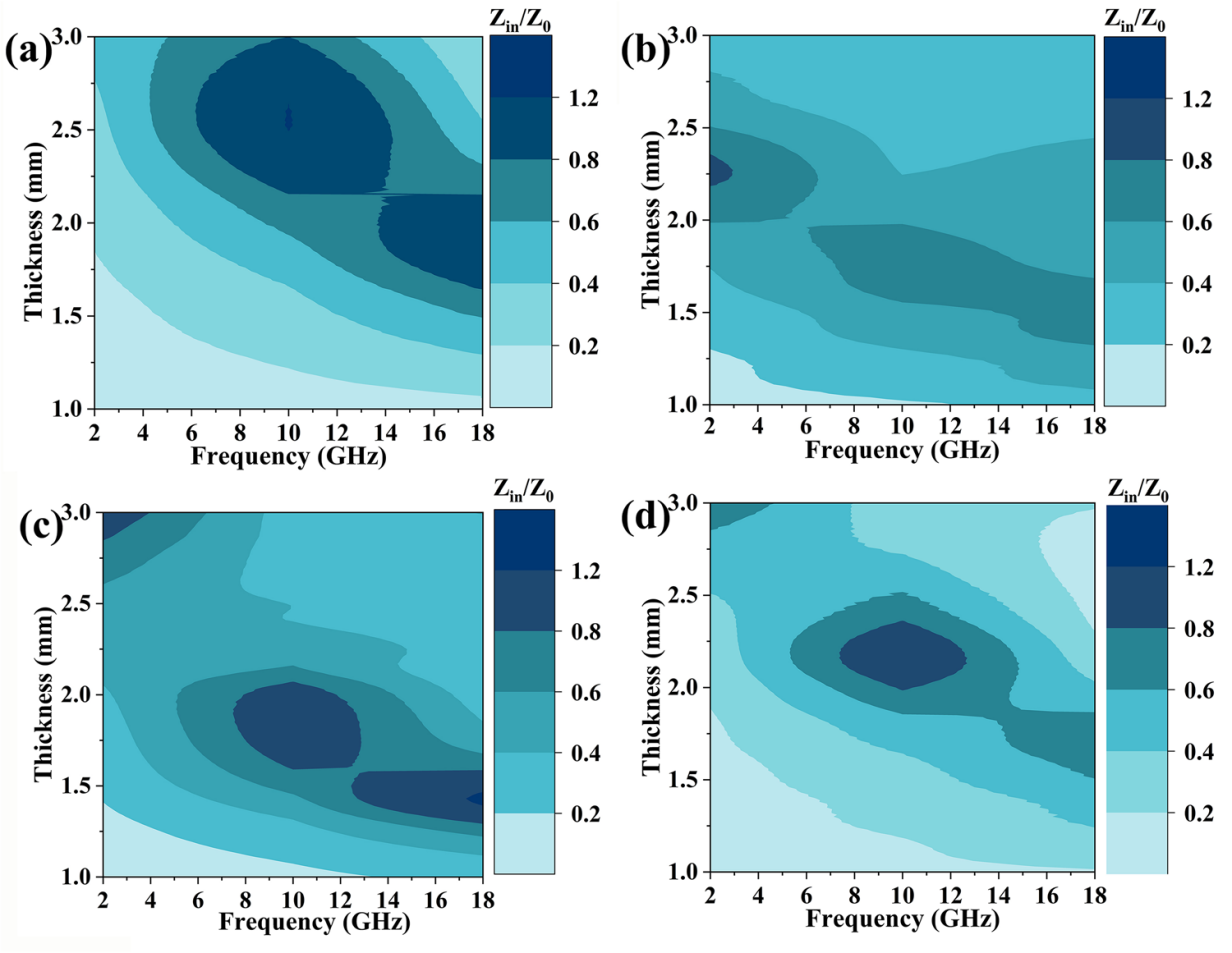
|*Z*_*in*_/*Z*_0_| of **a** FeCo/NC-600, **b** FeCo/NC-700, **c** FeCo/Fe_3_O_4_/NC-600, and **d** FeCo/Fe_3_O_4_/NC-700 for different frequencies and thicknesses

Typically, excellent impedance matching (|*Z*_*in*_*/Z*_*0*_|) is an essential prerequisite for a good EMW absorber. When |*Z*_*in*_*/Z*_*0*_| equals 1, the incident EMWs can penetrate the surface of the materials without undergoing any reflection. Figure 6 shows the contour maps of the |*Z*_*in*_*/Z*_*0*_| values for all the samples with a thickness of 1.0–3.0 mm at a frequency of 2–18 GHz. FeCo/NC-700 reaches the desired |*Z*_*in*_*/Z*_*0*_| value of 0.8–1.2 for a higher thickness range of 1.5–3.0 mm, while FeCo/Fe_3_O_4_/NC-600 achieves that at a lower thickness range of 1.2–2.1 mm.

Another important parameter for comprehensively analyzing EMW performance is attenuation constant (*α*), which can be calculated using the method mentioned in SI. When α = 169 and |*Z*_*in*_*/Z*_*0*_|= 1.00, FeCo/NC-700 aerogel exhibits an ultra-strong EMWA performance of − 85 dB, which indicates that more than 99.999999% of EMWs are absorbed by FeCo/NC-700 aerogel (Fig. S7a). For FeCo/Fe_3_O_4_/NC-600 aerogel, at a thickness of 1.59 mm, the |*Z*_*in*_*/Z*_*0*_| values fall in the range of 0.5–1 with good impedance matching, while the *α* values range from 150 to 335 and represent a strong EMW loss; thus, FeCo/Fe_3_O_4_/NC-600 aerogel achieves an ultra-broad *f*_*e*_ value of 7.44 GHz from 10.56 to 18.00 GHz (Fig. S7b).

**Fig. 7:**
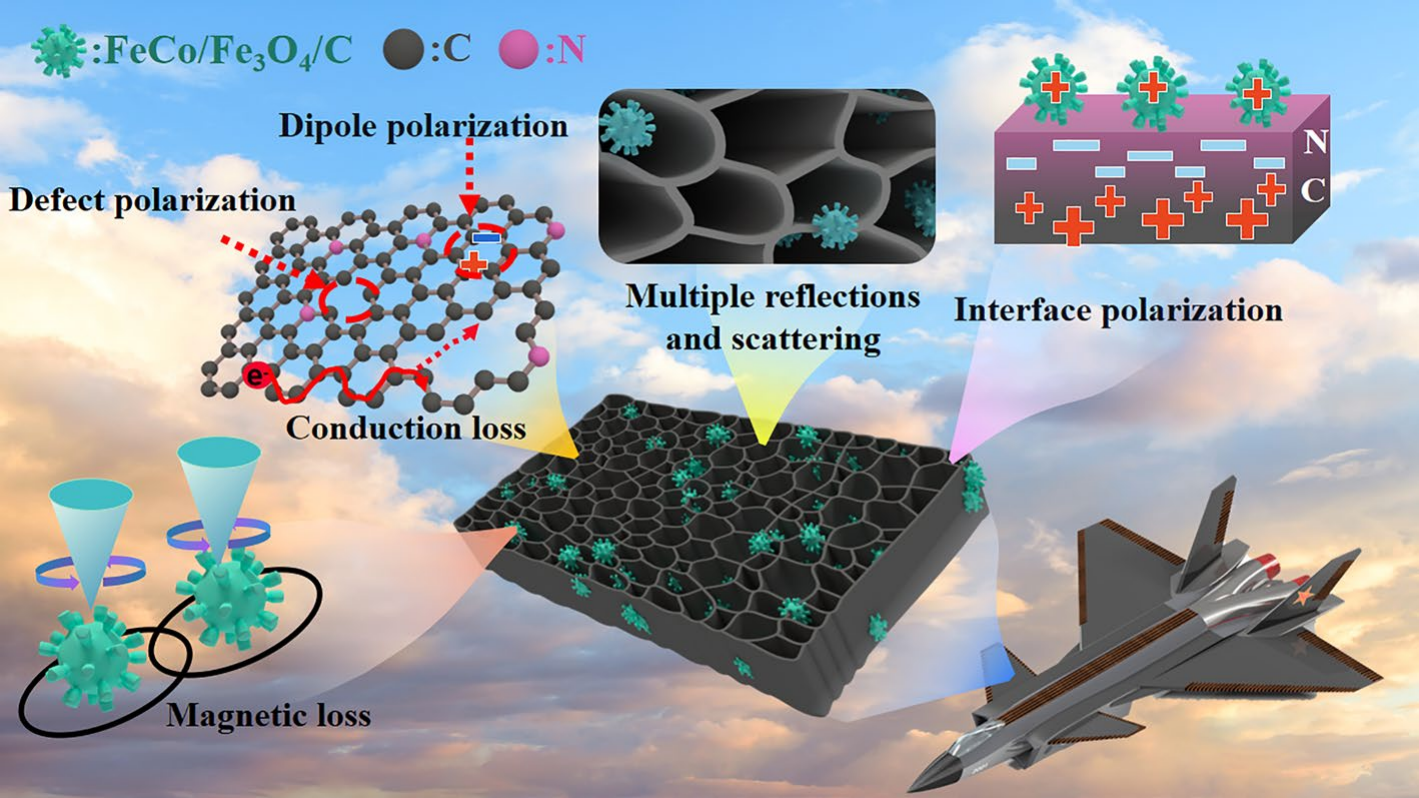
Schematic showing the EMWA mechanism of FeCo/Fe_3_O_4_/NC-600

Figure 7 shows that the EMW loss of FeCo/Fe_3_O_4_/NC aerogel mainly relies on impedance matching as well as the synergistic effect of conduction, polarization, and magnetic loss [36–38].

Aerogel pores not only facilitate the entry of EMWs into the material and thus balance the impedance, but also provide effective pathways for multiple reflection and scattering of EMW and thus increase the attenuation ability for EMWs [39, 40].

The conductive NC network embedded by magnetic particles is the main source of conduction losses. According to the electromagnetic induction theory, an alternating magnetic field forces free electrons to undergo directional migration, thus generating a microcurrent in the conducting network [37]. These electrons pass through the nanoparticles while causing violent collisions, thereby converting EMWs into heat energy. Along with ascending of the calcination temperature, the graphitization degree and conduction loss increase, which is proved by the long tails in the Cole–Cole plots of FeCo/NC-700 and FeCo/Fe_3_O_4_/NC-700 (Fig. S8).

**Fig. 8:**
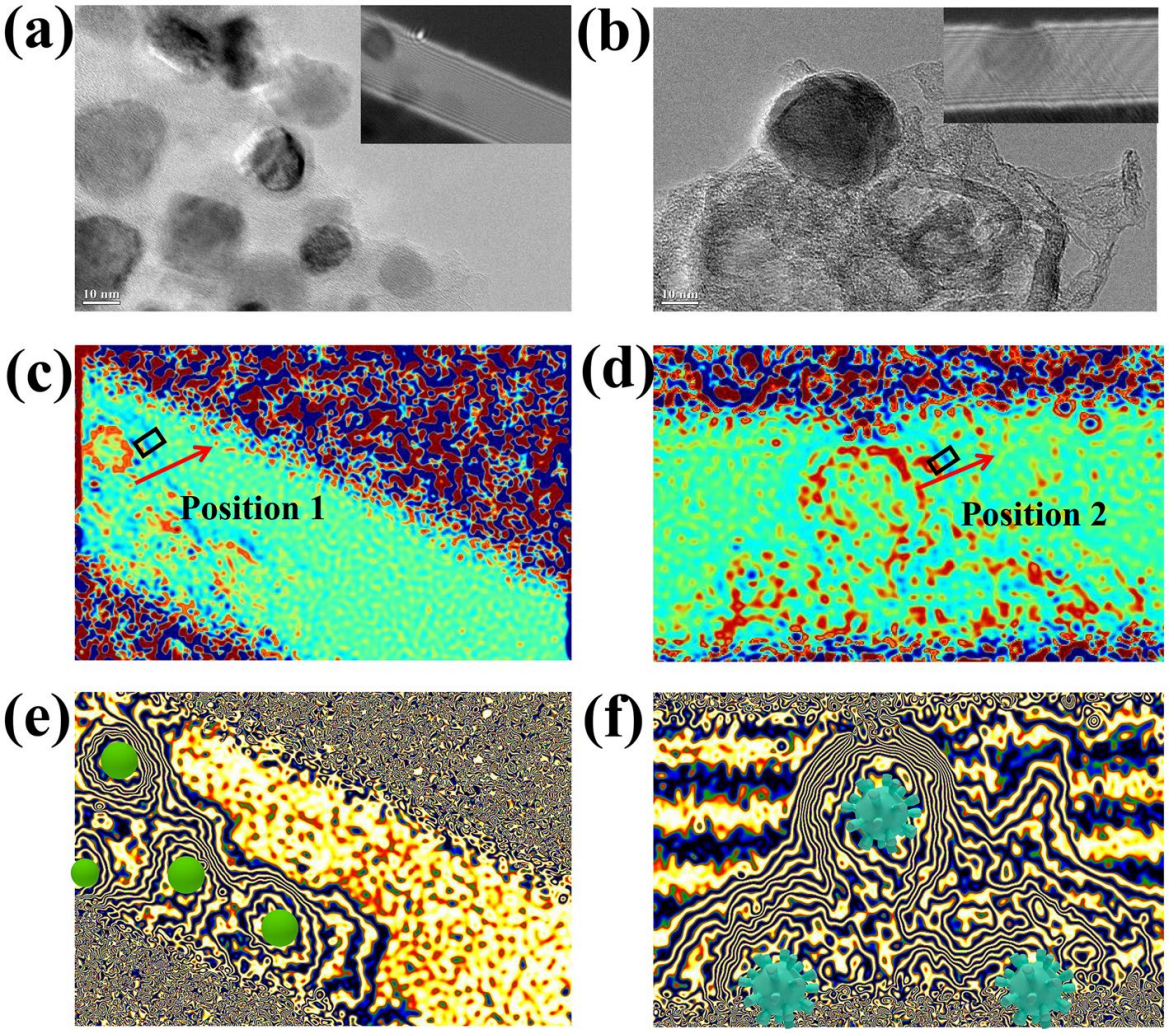
**a**, **b** TEM images and holograms, **c**, **d** charge density maps, **e**, **f** corresponding magnetic lines of flux of FeCo/NC-700, and FeCo/Fe_3_O_4_/NC-600, respectively

As shown in Fig. S8, the samples calcinated at 600 °C display more cole–cole semicircles than those calcinated at 700 °C, implying that these samples undergo more Debye relaxation processes and experience higher polarization losses [41, 42]. For FeCo-MOG/CP, the high-temperature calcination produces multitudinous defects in its derivatives, which is demonstrated by the XPS spectra; these defects lead to asymmetric charge distributions, thereby inducing defect polarization [31, 43]. Meanwhile, some functional groups in MOG and CP are retained, which act as dipole centers to generate dipole polarization [44]. Moreover, TEM images and electron holography reveal the presence of heterogeneous interfaces (Fig. 8a–f). Figure 8c–d shows that positive charges (marked in blue) are distributed on the carbon side and negative charges (marked in yellow) accumulate on the side of magnetic particles, which leads to micro-interfacial capacitance. Two inverse peaks can be observed in Fig. S9, showing that the variation in charge density from 0.37 to − 0.28 and 0.3 to − 0.43 e nm^−3^ occurs across the interfaces of the FeCo/NC and FeCo/Fe_3_O_4_/NC samples, respectively. Under a high-frequency electromagnetic field, the electric dipoles form due to opposite charges on different sides of the interface revolve repeatedly, thereby intensifying the interfacial polarization loss. All polarization losses promote dielectric loss and EMWA performance.

Figure S10 shows the magnetic hysteresis loops of FeCo/NC-600, FeCo/NC-700, FeCo/Fe_3_O_4_/NC-600, and FeCo/Fe_3_O_4_/NC-600 aerogels, thus revealing their saturation magnetization (*Ms*) to be 145, 123, 82, and 69 emu g^−1^, respectively. After the introduction of Fe_3_O_4_, the coercivities of aerogels significantly decrease, which facilitates the increase in permeability (Fig. S4). Meanwhile, the MOG causes nano-magnetic particles to be uniformly embedded on a three-dimensional carbon aerogel, thus avoiding the agglomeration of nano-magnetic particles. By analyzing the magnetic flux lines of FeCo/NC-700 in free space, it is observed that all the linearly arranged FeCo nanoparticles generate a special magnetic vortex structure due to their small size and couple with each other to form a dense magnetic network that passes through the carbon (Fig. 8e) [2]. At a nano-micro level, the magnetic induction lines of the two kinds of magnetic particles in the FeCo/Fe_3_O_4_/NC-600 aerogel form a big semicircle due to strong magnetic coupling (Fig. 8f). Remarkably, Fe_3_O_4_ shows a faster magnetic response than FeCo due to its lower coercivity under a high-frequency magnetic field, which in turn causes a higher relaxation loss after coupling with FeCo and enhances the hysteresis loss capability.

Additionally, the magnetic losses caused by ferromagnetic resonance and eddy current loss also contribute significantly to EMWA [45]. By analyzing the eddy current coefficient (*C*_*0*_) of the samples (Fig. S11), the existence of eddy current loss is proved by the scarce fluctuations in the *C*_*0*_ values from 6 to 14 GHz [46]. Some NC aerogels with low graphitization degree restrain the agglomeration of magnetic particles and cut off the eddy current, and consequently the skin effect is suppressed effectively and the magnetic functions of isolated Fe_3_O_4_ and FeCo are developed fully [47]. Fluctuation in *C*_*0*_ values from 2–6 GHz and 14–18 GHz occur due to natural and exchange resonance, respectively, which indicates that a high natural resonance takes effect because of the strong anisotropy of magnetic particles in the aerogels [48].

Here, similar to how viruses exhibit intense pathogenicity, virus-shaped particles also show strong functionality. The small spikes on the surface act like antennas and can improve the ability to absorb and transmit EMWs. Meanwhile, analogous to ribonucleic acid, CP with its helical structure can produce cross-polarization [49, 50]; thus, both virus-shaped particles and CP can enhance the loss of EMWs. The virus-shaped particles with a large size also exhibit a customized dual-soft-magnetic effect that enlarges the magnetic network from nanometer scale to centimeter scale, which makes FeCo/Fe_3_O_4_/NC-600 more suitable for centimeter waves and facilitates the loss of EMWs [51]. Owing to various EMWA mechanisms, FeCo/NC-700 achieves an ultra-strong absorption intensity of − 85 dB at an ultra-low load ratio of 5% and FeCo/Fe_3_O_4_/NC-600 demonstrates an ultra-wide absorption band of 7.44 GHz at a thickness of 1.55 mm.

### Thermal Insulation Performance

In the current complex international situation, the heat generated by stealth fighters and missiles during flight causes them to be easily detected and tracked using infrared detection technology; thus, an excellent thermal insulation performance combined with good EMWA capability can lead to perfect stealth properties [52, 53].

**Fig. 9:**
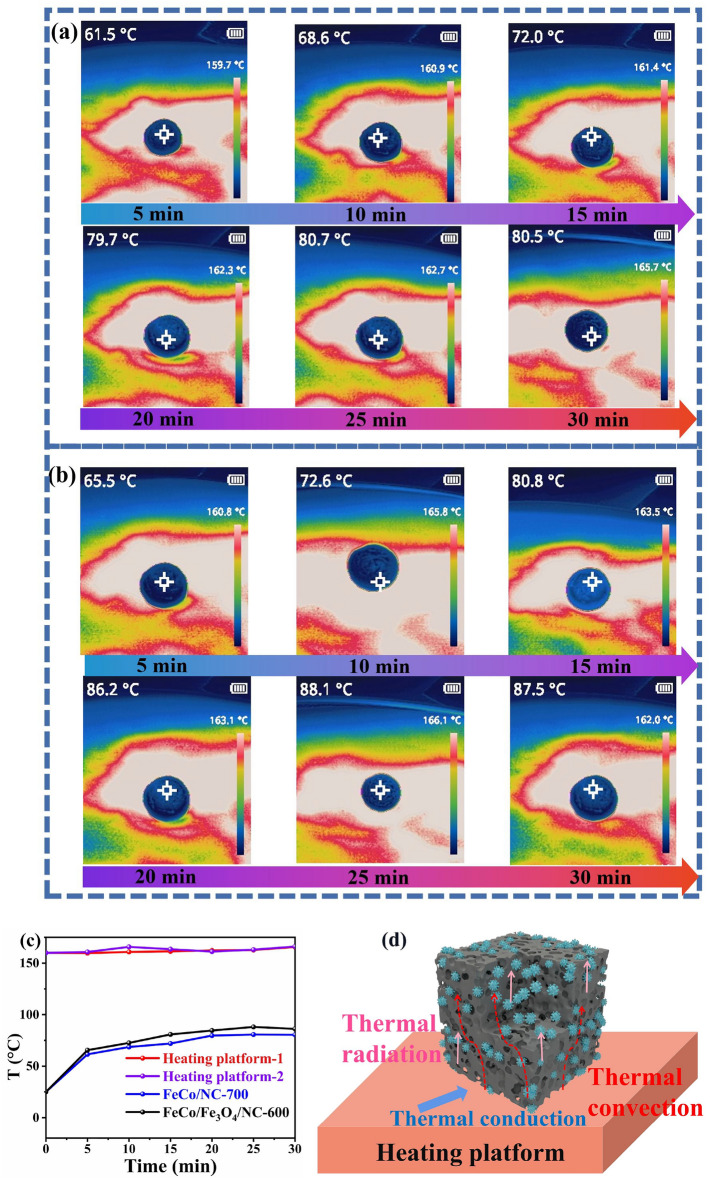
Thermal infrared images of **a** FeCo/NC-700 and **b** FeCo/Fe_3_O_4_/NC-600 at 180 °C; **c** temperature variation curve; **d s**chematic of the heat transfer mechanism of samples

Thermal energy is mainly transferred via conduction, convection, and radiation (Fig. 9). Thermal conduction is mainly implemented by direct contact of solid materials. Since the aerogel comprises excess air with a relatively low thermal conductivity, the heat at the bottom of aerogel cannot be transferred to the upper surface. Moreover, the tortuous path of air flow in the aerogel network reduces thermal convection, and the solid–gas interface dissipates the thermal radiation energy via reflection and scattering. The thermal conductivities of FeCo/NC-700 and FeCo/Fe_3_O_4_/NC-600 reach 0.043 and 0.049 W m^−1^ K^−1^, respectively. To test the thermal insulation performance of FeCo/NC-700 and FeCo/Fe_3_O_4_/NC-600, we place them on the heating platform for 5 min at the temperatures of 180 °C and then record the temperature change. The thermal imaging camera records that the temperatures of the two samples are much lower than those of the heating platform; thus, the aerogels exhibit a good thermal insulation performance. Furthermore, due to the large temperature difference between the sample and heating platform, the temperature of the sample rises rapidly within 0–5 min and then stabilizes at around 25 min. The temperatures of FeCo/NC-700 and FeCo/Fe_3_O_4_/NC-600 remain stable at about 80.9 and 87.5 °C, respectively. Because FeCo/NC-700 is more porous (Fig. 2), the thermal insulation performance of FeCo/NC-700 is slightly better than that of FeCo/Fe_3_O_4_/NC-600.

### Radar Stealth Performance

**Fig. 10:**
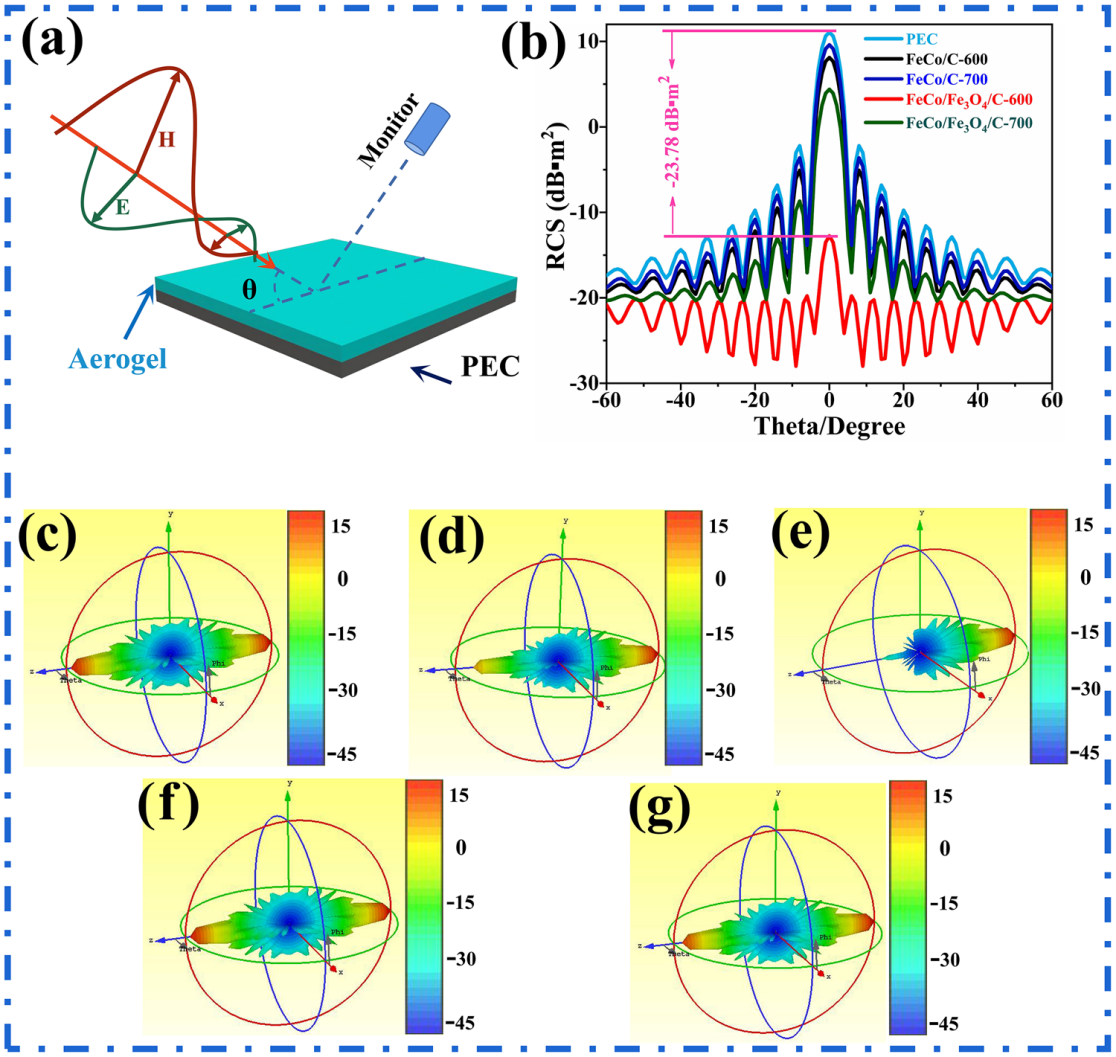
**a** Sketch of the CST simulation, **b** RCS curves for square-shaped models, and three-dimensional RCS results for square-shaped models coated with **c** PEC **d** FeCo/NC-600, **e** FeCo/NC-700, **f** FeCo/Fe_3_O_4_/NC-600, and **g** FeCo/Fe_3_O_4_/NC-700

To analyze the radar stealth performance of samples, the radar cross section (RCS) of models coated with a perfect electric conductor (PEC) and all the other samples are simulated under real far-field conditions using CST [54–56]. Figure 10a shows that a square-shaped (20 × 20 cm^2^) PEC model is coated with aerogel samples and PEC to produce five models with a coating thickness of 2.44 mm. The five models are placed in the XY plane and subjected to parallel 7.52 GHz EMW irradiation. The angle (θ) between the EMWs and the Z-axis is varied from − 90° to 90°. Figure 10b shows that when θ is 0°, the incident EMWs are directly perpendicular to the models and the difference in the RCS values of the models coated with FeCo/Fe_3_O_4_/NC-600 and PEC is − 23.78 dB m^2^, which proves that FeCo/Fe_3_O_4_/NC-600 has excellent radar stealth performance. Furthermore, the RCS values of the model coated with PEC is relatively larger than those of the model coated with the aerogel samples for the three-dimensional RCS results (Fig. 10c–g), which demonstrates that all samples have the ability to attenuate radar waves.

**Fig. 11:**
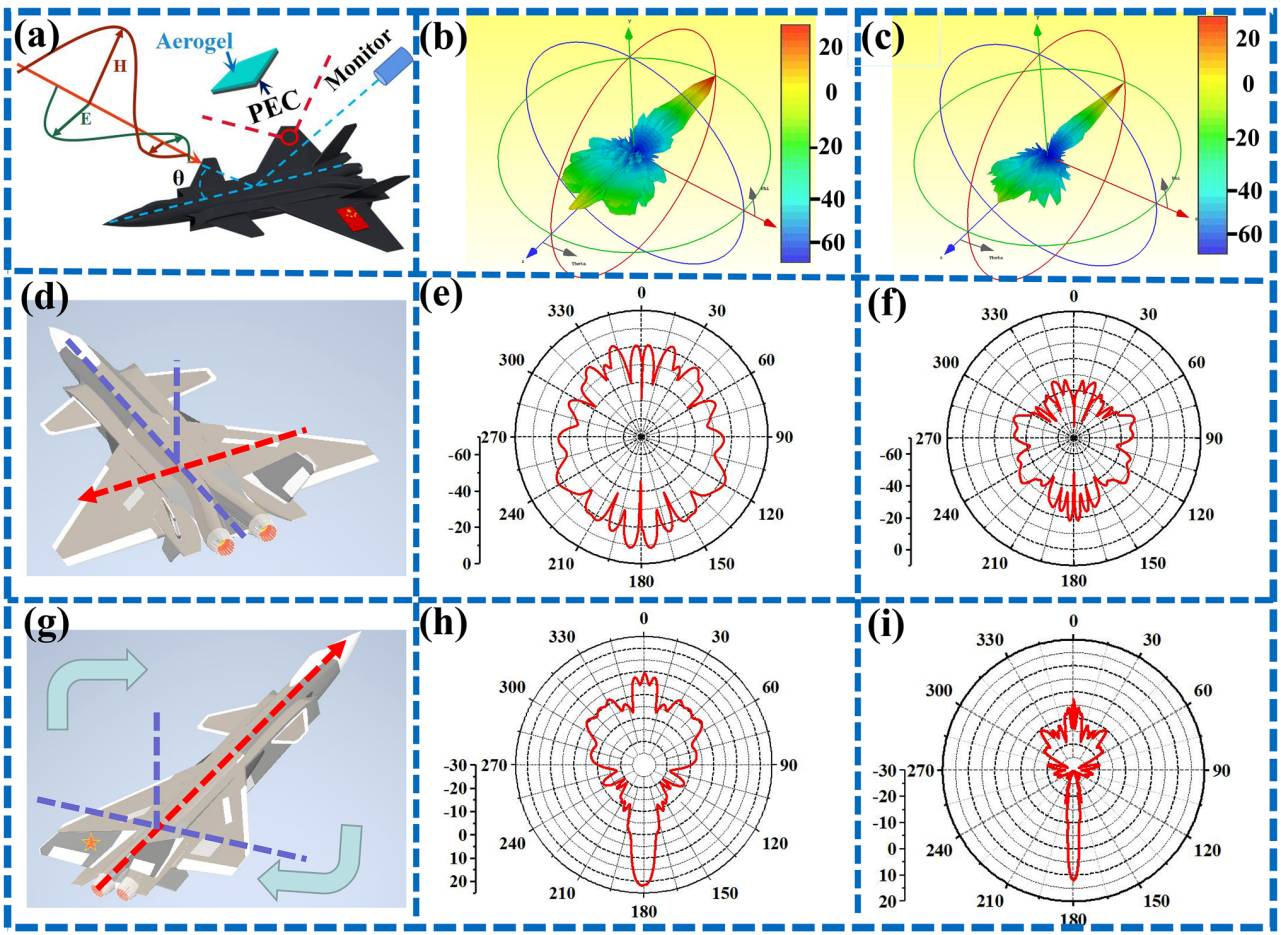
**a** Sketch representing the CST simulation of the J-20 fighter model coated with aerogel, three-dimensional RCS results of the J-20 fighter models coated with **b** PEC, and **c** FeCo/Fe_3_O_4_/NC-600, RCS results of J-20 fighter models coated with **d**–**f** FeCo/Fe_3_O_4_/NC-600 laterally, and **g**–**i** longitudinally

Meanwhile, we also construct a J-20 fighter model of to simulate the RCS of the FeCo/Fe_3_O_4_/NC-600 aerogel for military applications. As shown in Fig. 11a, the FeCo/Fe_3_O_4_/NC-600 aerogel with a thickness of 2.44 mm is coated on the J-20 fighter model comprising PEC, after which the radar stealth performance of FeCo/Fe_3_O_4_/NC-600 is tested by irradiating the J-20 model with parallel EMWs of 7.52 GHz. Figure 11b–c shows that the RCS values of J-20 fighter coated with the FeCo/Fe_3_O_4_/NC-600 aerogel are significantly lower than those of the uncoated model. By simulating the RCS of J-20 fighter laterally (Fig. 11d–f) and longitudinally (Fig. 11g–i), it is found that FeCo/Fe_3_O_4_/NC-600 materials can effectively reduce the RCS values of the J-20 fighter, especially those of its wings.

## Conclusions

In summary, this is the first study that has developed two types of magnetic NC aerogels by using MOG as the precursor. Inheriting the features of FeCo-MOG/CP, the magnetic particles are uniformly dispersed on the NC matrix of the obtained aerogels, and the analyses of the heterometallic magnetic coupling behavior of the samples demonstrate their excellent magnetic loss capability. The multi-polarization loss generated via nitrogen doping on the carbon skeleton and the embedding of FeCo and Fe_3_O_4_ on the NC matrix provide a synergistic effect and increase the EMW attenuation. At a heating rate of 2 °C min^−1^, FeCo/NC aerogel is obtained, which achieves an ultra-strong EMWA performance of − 85 dB at an ultra-low loading of 5%. The Fe_3_O_4_ magnetic nanoparticles with semiconducting characteristics and low coercivity help achieve better impedance matching and reduce the matching thickness of absorbers. Thus, when we increase the heating rate to 5 °C min^−1^, FeCo/Fe_3_O_4_/NC aerogels are obtained, which possess a wide absorption band of 7.44 GHz at an ultra-thin thickness of 1.59 mm. The aerogel comprises excess air and exhibits an excellent thermal insulation property. Furthermore, the excellent radar stealth performance of the aerogels is verified by conducting CST simulation under real far-field conditions. Overall, utilizing MOG as precursors is a pioneering path for the preparation of light, uniform, and stable inorganic–organic hybrid aerogels; thus, this study is expected to provide considerable ideas for developing multi-functional materials with excellent thermal insulation, EMWA capability, and radar stealth performance.

## Supplementary Information

Below is the link to the electronic supplementary material.

Supplementary file1 (PDF 2828 KB)

Supplementary file2 (MP4 36967 KB)
