# Supplementary material for: Metal–Organic Gel Leading to Customized Magnetic-Coupling Engineering in Carbon Aerogels for Excellent Radar Stealth and Thermal Insulation Performances
Source: Nanomicro Lett. 2023 Dec 4;16:42. doi: 10.1007/s40820-023-01255-7 (PMC10695913; doi:10.1007/s40820-023-01255-7)
Supplement: Supplementary file 1 — Supplementary file1 (PDF 2828 KB) [file 40820_2023_1255_MOESM1_ESM.pdf]

Supporting Information for

Metal-Organic Gel Leading to Customized Magnetic-Coupling Engineering in Carbon Aerogels for Excellent Radar Stealth and Thermal Insulation Performances

Xin Li[1,2], Ruizhe Hu[1,2], Zhiqiang Xiong[2], Dan Wang[1,2], Zhixia Zhang[1,2], Chongbo Liu[1,2,]*, Xiaojun Zeng[4], Dezhi Chen[1,2], Renchao Che[3,*], Xuliang Nie[5,*]

[1] Key Laboratory of Jiangxi Province for Persistent Pollutants Control and Resources Recycle, Nanchang Hangkong University, Nanchang 330063, P. R. China

[2] School of Environmental and Chemical Engineering, Nanchang Hangkong University, Nanchang 330063, P. R. China

[3] Laboratory of Advanced Materials, Department of Materials Science and Collaborative Innovation Center of Chemistry for Energy Materials and Collaborative Innovation Center of Chemistry for Energy Materials, Fudan University, Shanghai 200438, P. R. China

[4] School of Materials Science and Engineering, Jingdezhen Ceramic University, Jingdezhen 333403, P. R. China

[5] College of Chemistry and Materials, Jiangxi Agricultural University, Nanchang, 330045, P. R. China

*Corresponding authors. E-mail: cbliu2002@163.com (Chongbo Liu); rcche@fudan.edu.cn (Renchao Che); xuliangnie123@163.com (Xuliang Nie)

S1 Experiment and Calculation Method

To perform microwave absorption measurements, the melted paraffin was evenly dipped into the sample through a vacuum-assisted impregnation method and molded into toroidal-shaped test samples (Φ_{out}: 7.0 mm, Φ_{in}: 3.04 mm). The densities of FeCo/NC and FeCo/Fe_3O_4/NC were 0.049 g·cm^{-3} and 0.052 g·cm^{-3}, respectively. The loading content of aerogels in paraffin can be calculated as follows:

$$W\%=m_1/(m_1+m_2) \quad \text{(S1)}$$

where m_1 and m_2 represent the aerogel and paraffin weights, respectively.

The RL values were calculated based on the transmission line theory by employing the following electromagnetic parameters:

$$RL = 20\lg\left|\frac{Z_{in}-Z_0}{Z_{in}+Z_0}\right| \quad \text{(S2)}$$

$$\left\{ Z_0=\sqrt{\frac{\mu_0}{\varepsilon_0}} \qquad Z_{in}=\sqrt{\frac{\mu_r}{\varepsilon_r}}tanh\left(j\frac{2\pi fd}{c}\sqrt{\mu_r\varepsilon_r}\right) \right\} \quad \text{(S3)}$$

where c is the velocity of light; f represents the microwave frequency; d is the absorber thickness; ε_r and μ_r are the relative complex permittivity and permeability, respectively; Z_0 is the impedance of free space; and Z_{in} is the input impedance of the absorber.

The thickness and diameter of aerogels tested for the thermal insulation performance are ca. 5.6 and ca. 19.5 mm respectively.

S2 Supplementary Results and Discussion

S2.1 High-angle Annular Dark-field TEM and Element Distribution Maps

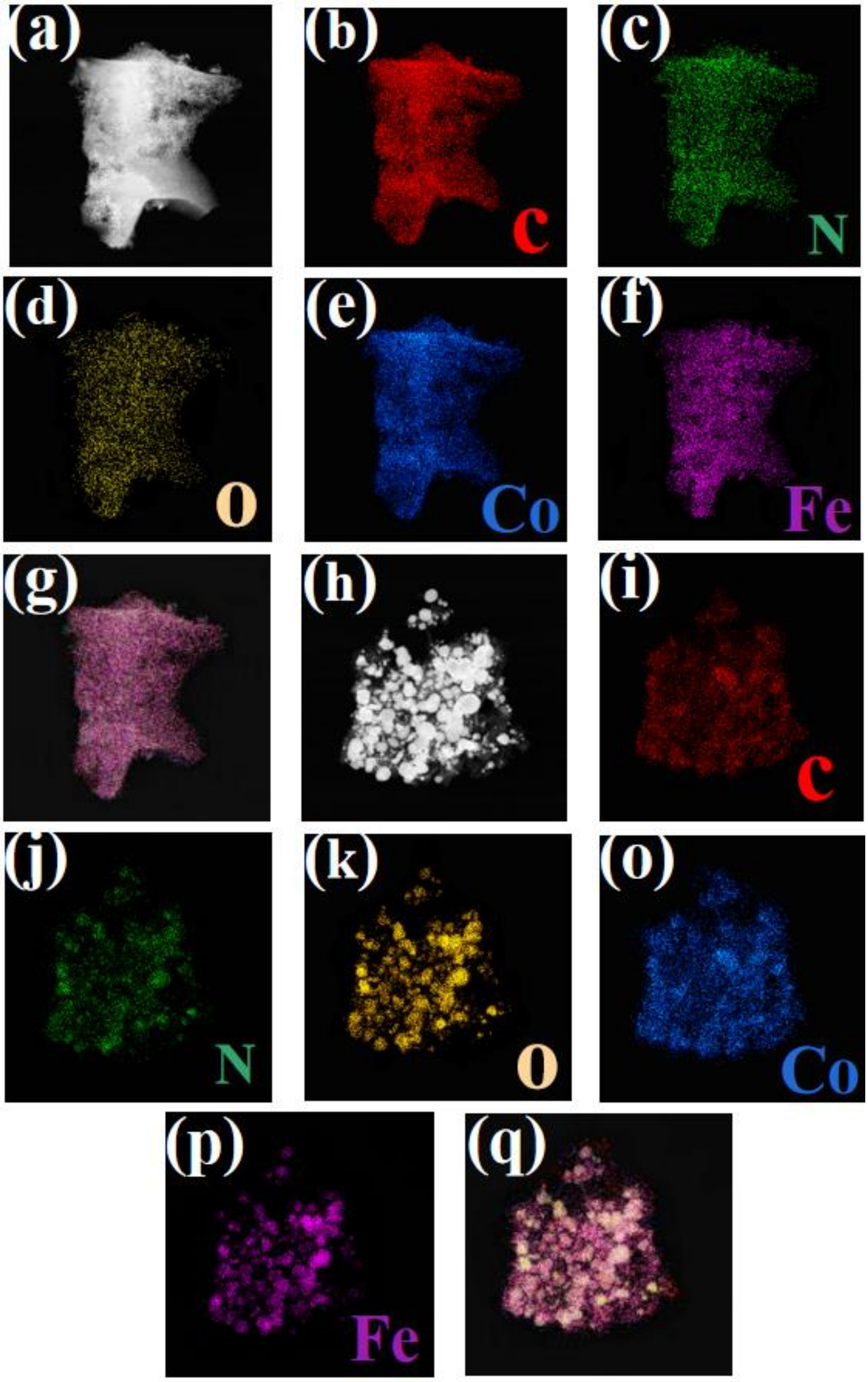

Fig. S1 The high-angle annular dark-field TEM and element distribution maps for **a**, **b**, **c**, **d**, **e**, **f, g** FeCo/NC, and **h**, **i**, **j**, **k**, **o**, **p,q** FeCo/Fe_3O_4/NC

S2.2 PXRD of FeCo-MOG/CP

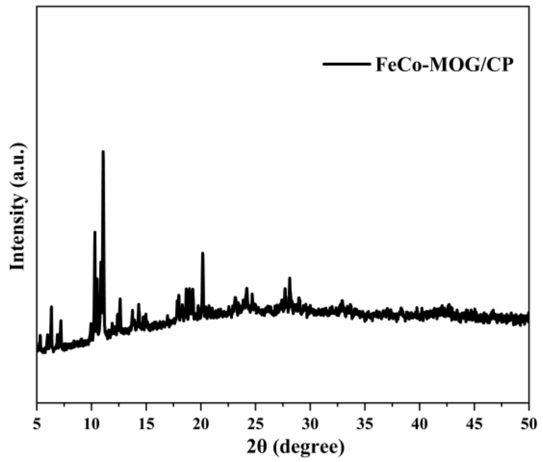

Fig. S2 The PXRD of FeCo-MOG/CP

S2.3 XPS

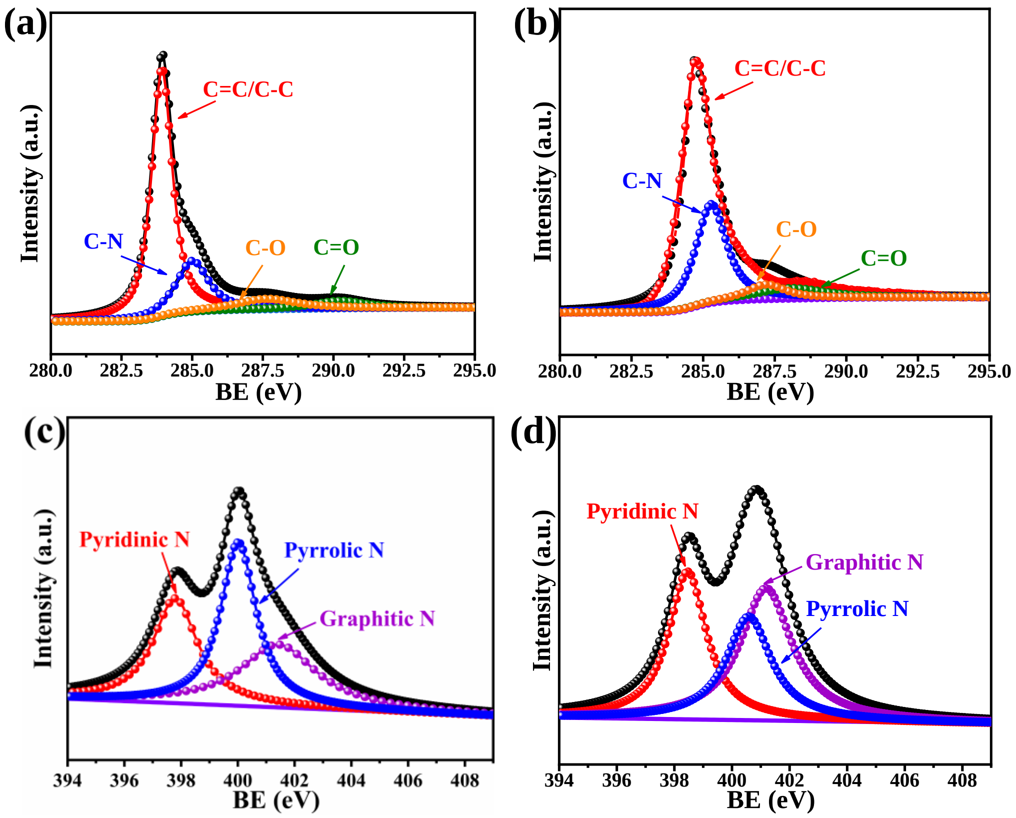

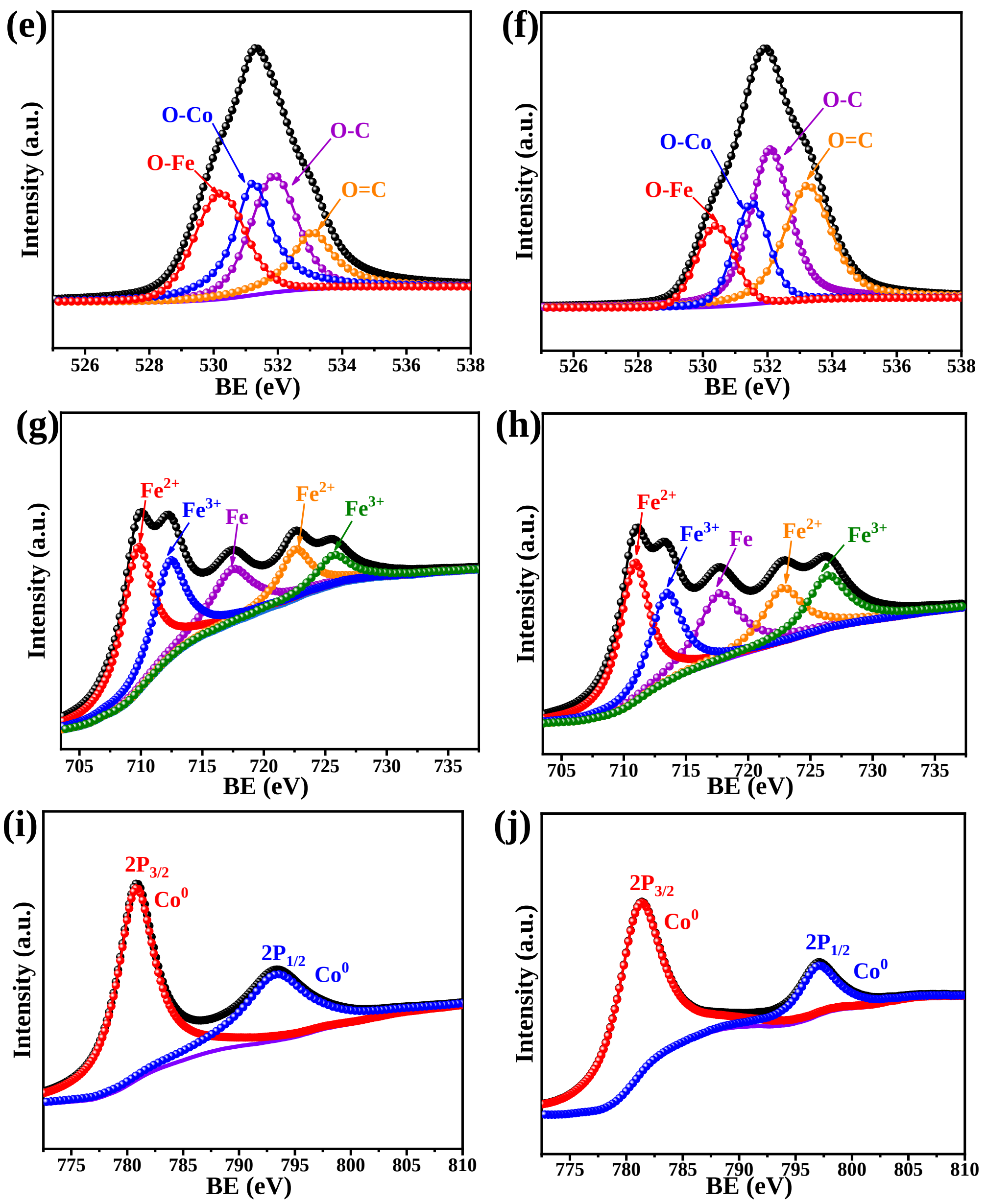

Fig. S3 The high-resolution spectra of **a**, **b** C 1S, **c-d** N 1S, **e, f** O 1S, **g, h** Fe 2p and **i, j** Co 2p for FeCo/NC and FeCo/Fe_3O_4/NC, respectively

S2.4 Electromagnetic Parameters

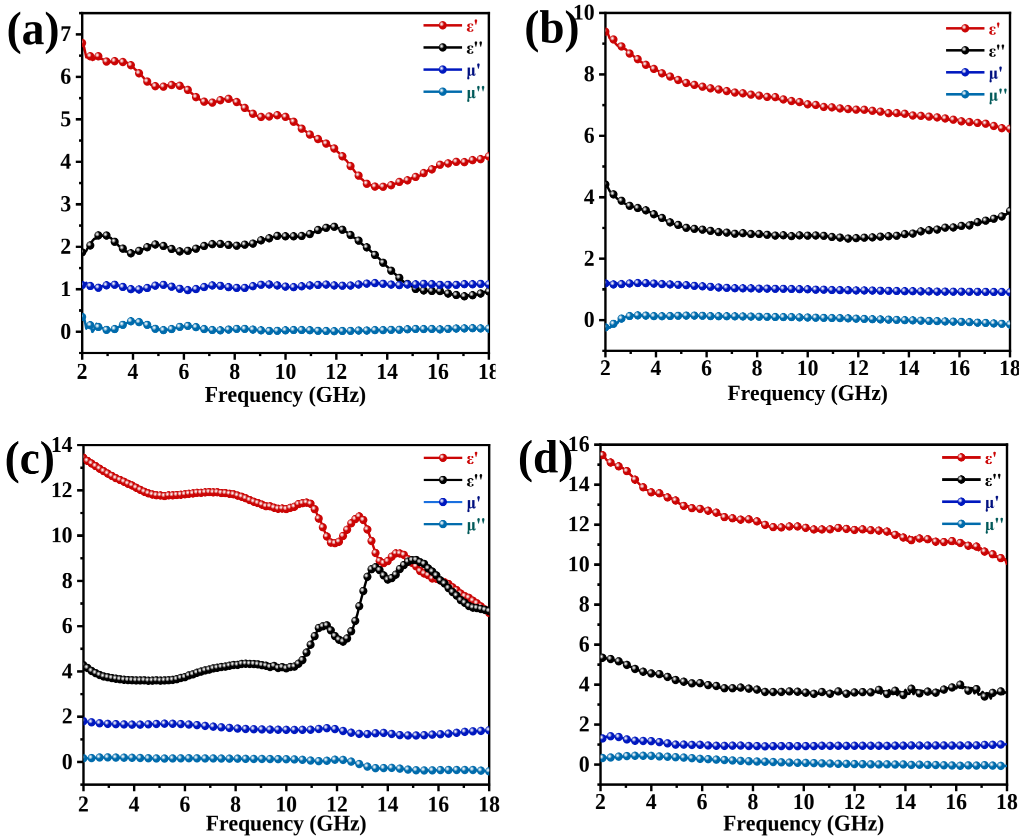

Fig. S4 The electromagnetic parameters of **a** FeCo/NC-600, **b** FeCo/NC-700, **c** FeCo/Fe_3O_4/NC-600, **d** FeCo/Fe_3O_4/NC-700

S2.5 Dielectric Loss Tangent and Magnetic Loss Tangent

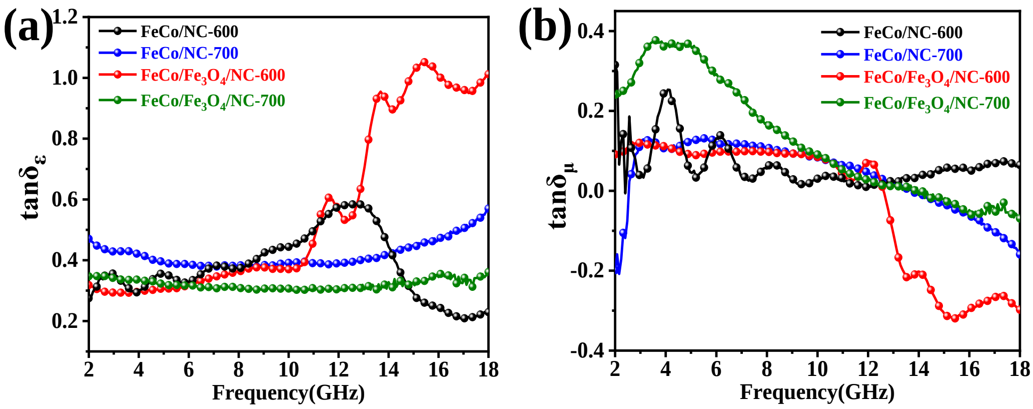

Fig. S5 The dielectric loss tangent **a** and magnetic loss tangent **b** of samples

S2.6 Frequency Dependence of Variation of RL Values and Simulation Thicknesses

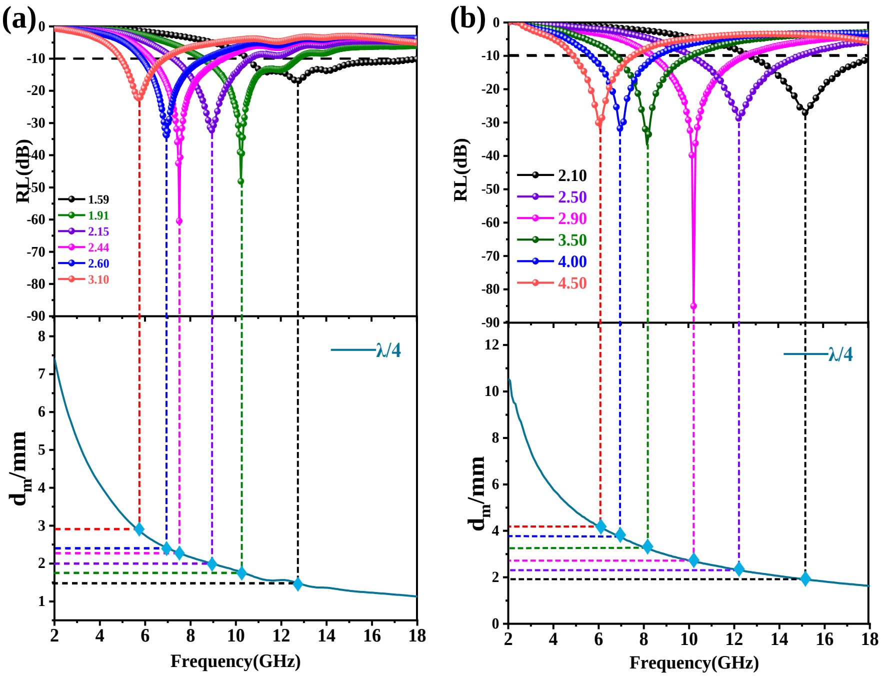

Fig. S6 Frequency dependence of variation of *RL* values and simulation thicknesses

S2.7 The relationship of Frequency, $|Z_{in}/Z_0|$ values, *α* and *RL* values

The attenuation constant (α) values can be expressed by the following equation:

$$\alpha = \frac{\sqrt{2}}{c}\pi f \times \sqrt{(\mu''\varepsilon'' - \mu'\varepsilon') + \sqrt{(\mu''\varepsilon'' - \mu'\varepsilon')^2 + (\mu'\varepsilon'' + \mu''\varepsilon')^2}} \quad \text{(S4)}$$

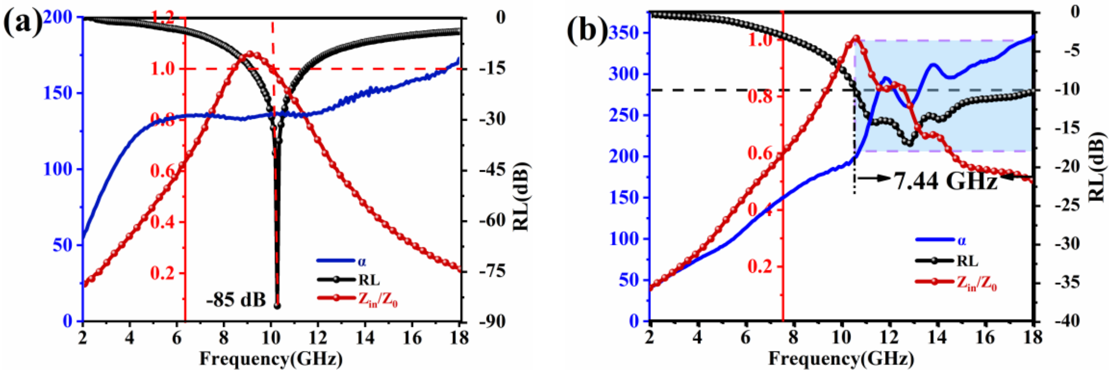

Fig. S7 Frequency dependence of the $|Z_{in}/Z_0|$ values, α and *RL* values of **a** FeCo/NC-700 at 2.90 mm, and **b** FeCo/Fe_3O_4/NC-600 at 1.59 mm

S2.8 Cole-cole Plots

Based on the Debye polarization theory, the relationship between ε' and ε'' can be described as follows:

$$(\varepsilon' - \frac{\varepsilon_s + \varepsilon_\infty}{2})^2 + (\varepsilon'')^2 = (\frac{\varepsilon_s - \varepsilon_\infty}{2})^2 \qquad \text{(S5)}$$

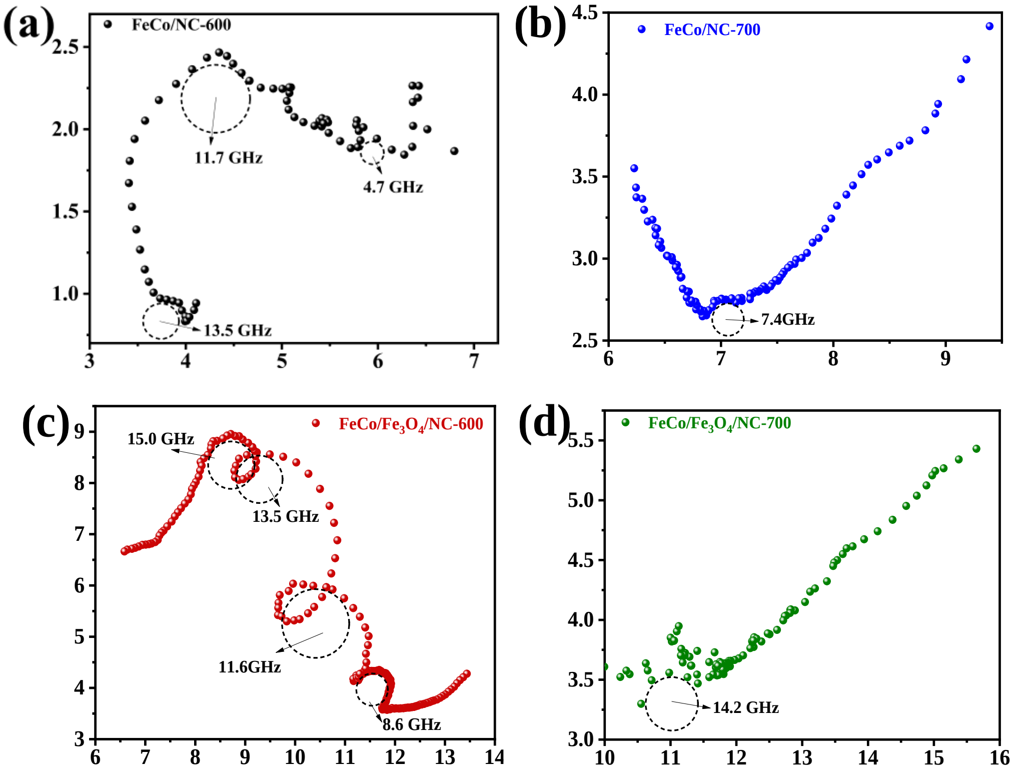

Fig. S8 Cole-cole plots (ε'' vs. ε') for all the samples

S2.9 Charge Distribution in the Direction of Arrows at Positions 1 and 2

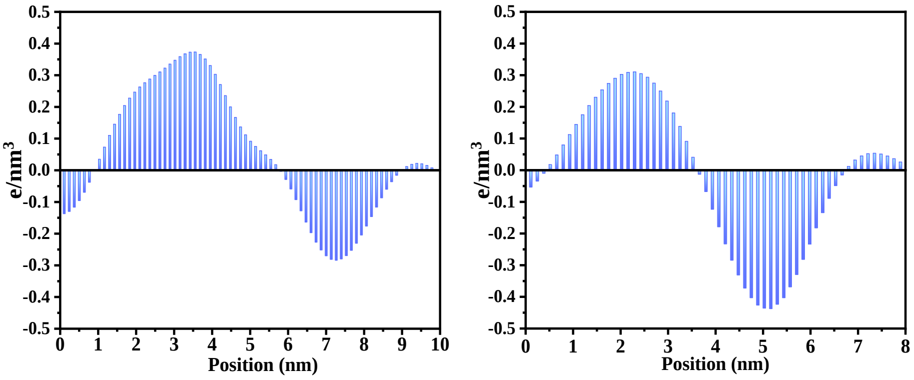

Fig. S9 The charge density maps of **a** position 1 of FeCo/NC, and **b** position 2 of FeCo/Fe_3O_4/NC

S2.10 Magnetic Hysteresis Loops

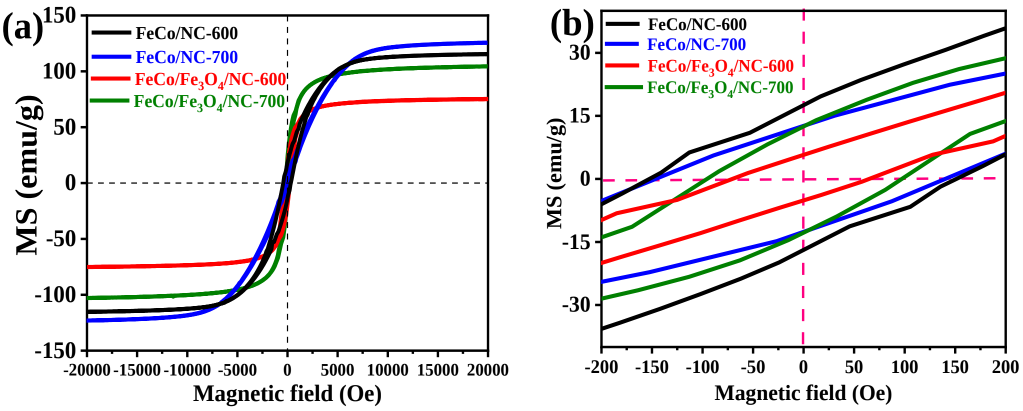

Fig. S10 The magnetic hysteresis loops of the samples

S2.11 Eddy Current Coefficient

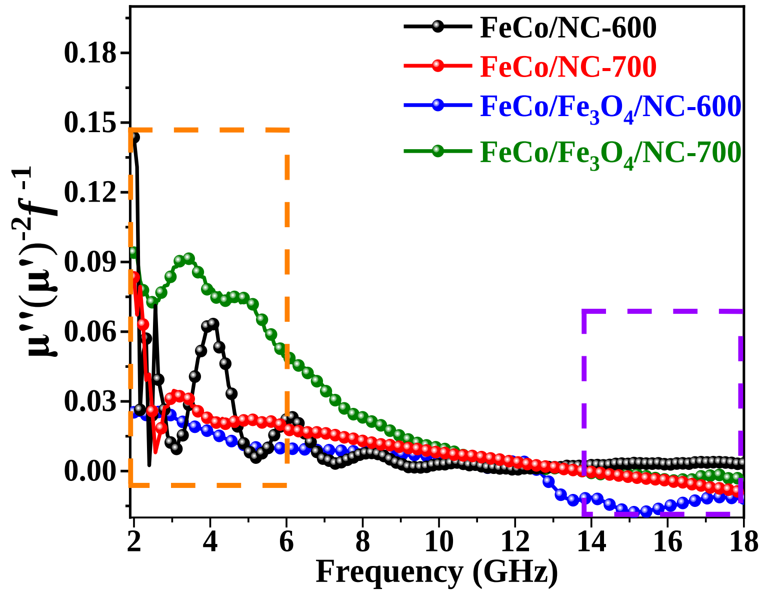

Fig. S11 The eddy current coefficient of the samples
